# Development of an Automatic Polarization Raman LiDAR for Aerosol Monitoring over Complex Terrain

**DOI:** 10.3390/s19143186

**Published:** 2019-07-19

**Authors:** Longlong Wang, Samo Stanič, William Eichinger, Xiaoquan Song, Marko Zavrtanik

**Affiliations:** 1 Center for Atmospheric Research, University of Nova Gorica, 5270 Nova Gorica, Slovenia; 2 Department of Civil and Environmental Engineering, University of Iowa, Iowa City, IA 52242, USA; 3 Ocean Remote Sensing Institute, Ocean University of China, Qingdao 266100, China; 4 Laboratory for Regional Oceanography and Numerical Modeling, Qingdao National Laboratory for Marine Science and Technology, Qingdao 266237, China; 5 Jožef Stefan Institute, 1000 Ljubljana, Slovenia

**Keywords:** polarization Raman LiDAR, system calibration, performance, complex terrain

## Abstract

High temporal and spatial resolution profiling of aerosol properties is required to study air pollution sources, aerosol transport, and features of atmospheric structures over complex terrain. A polarization Raman LiDAR with remote operation capability was developed for this purpose and deployed in the Vipava Valley, Slovenia, a location in the Alpine region where high concentrations of aerosols originating from a number of different local and remote sources were found. The system employs two high-power Nd:YAG pulsed lasers at 355 nm and 1064 nm as transmitters and provides the capability to extract the extinction coefficient, backscatter coefficients, depolarization ratio, Ångström exponent, and LiDAR ratio profiles. Automatized remote operation in an indoor environment provides a high duty cycle in all weather conditions. In addition to the detailed description of the device, an assessment of its potential and the retrieval uncertainties of the measured quantities is discussed. System optimization and performance studies include calibration of the depolarization ratio, merging of near-range (analog) and far-range (photon counting) data, determination of overlap functions, and validation of the retrieved observables with radiosonde data. Two cases for assessing LiDAR performance under specific weather conditions (during rain and in the presence of mineral dust) are also presented.

## 1. Introduction

**Figure 1:**
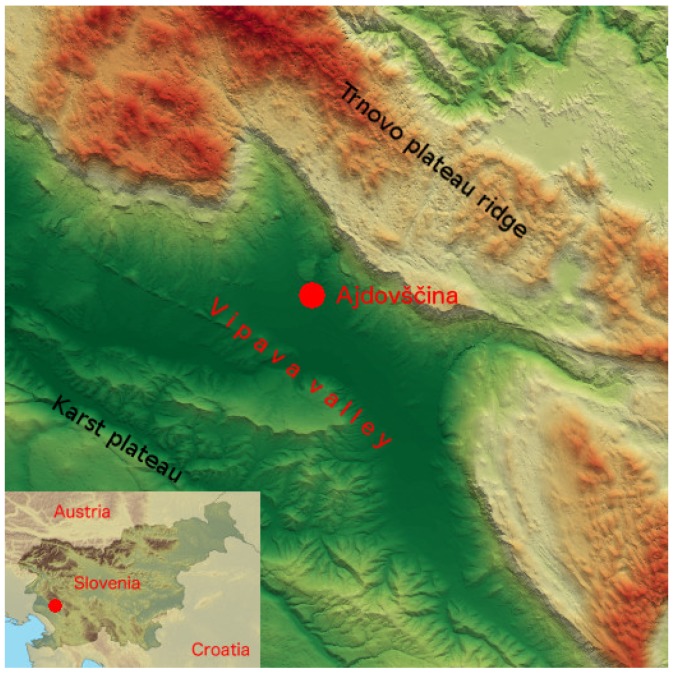
Topographic features of the Vipava Valley, located in the southwestern part of Slovenia, approximately 25 km from the Adriatic coast (inlay). On its southern side, it is confined by the Karst plateau (up to 300 m a.s.l.) and on its northern side by the steep Trnovo plateau ridge, which rises up to about 1000 m a.s.l (http://gis.arso.gov.si). LiDAR was deployed at Ajdovščina (45.87${}^{\circ }$ N, 13.90${}^{\circ }$ E, 125 m a.s.l.).

Mountainous areas with a dense population may contain aerosol sources with poorly-explored aerosol dispersion mechanisms, which significantly contribute to air pollution on local and regional scales [1]. The main reasons for the increased air pollution over complex terrain are limited vertical turbulent mixing in calm weather, which is in general more complicated than over a plane, and high local densities of aerosol sources in mountain valleys [2,3]. Vertical mixing and horizontal aerosol transport are also affected by downslope winds and accompanying periodic phenomena such as mountain waves and Kelvin–Helmholtz waves [4,5]. A representative air pollution hot-spot in an Alpine region is the Vipava Valley in southwestern Slovenia. As it is surrounded by mountains from three sides (Figure 1), stable atmospheric conditions lead to the formation of strong vertical aerosol gradients in the lower troposphere up to the height of the encircling mountain ranges. Being only 30 km away from Adriatic coast, the area is frequently affected by long-range transport of mineral dust from North Africa, as well as by sea salt aerosols from the Adriatic and the Mediterranean. Local emissions of anthropogenic aerosols, which mainly originate from biomass burning and traffic, cause heavy air pollution within the valley due to their weak diffusion, especially in the winter [6], which is dispersed either by strong precipitation or wind, especially by frequent onsets of cold, strong, and gusty downslope wind called Bora [7], which is capable of inducing circulations that lift particulate matter and trace gases into the free troposphere [5]. In order to monitor aerosol dispersion in the environment and investigate atmospheric structures that arise from wind and orographically-generated waves, high temporal and spatial resolution profiling of aerosol properties is needed. Continuous LiDAR profiling of those aerosol properties, which can reveal the predominant aerosol type, is mandatory to identify aerosol sources and understand dispersion mechanisms. In addition to the information from in situ meteorological and pollution monitoring (i.e., of black carbon), the information on sources and dispersion mechanisms is a necessary input for setting air pollution mitigation policy. Continuous LiDAR profiling in all weather conditions is also the key to revealing the dynamics of poorly-understood atmospheric phenomena, such as Bora [5,8].

The requirements regarding high temporal and spatial resolution of data, as well as aerosol identification capabilities can be met by advanced ground-based LiDAR systems with multiple detection channels [9,10,11,12,13,14,15], which can obtain a broad set of information on physical processes and optical properties in the atmosphere in the presence of different aerosol types. The majority of these systems currently in operation are manually-operated prototypes, differing considerably from one device to another, which leads to a variety of system setups with very different features and data quality [16,17]. To be able to compare data originating from similar processes at different locations, a detailed understanding of the performance of each system is required, and a thorough assessment of the uncertainties of the retrieved quantities needs to be provided. Furthermore, robotization of LiDAR devices is also very important to decrease data variability due to manual operation and thus increase their reliability in continuous long-term observations [18]. Our goal was to develop an automatic LiDAR system with high accuracy for long-term monitoring of atmospheric structures and a broad set of aerosol optical properties, such as aerosol backscatter coefficients [19,20,21], extinction coefficient [20,21], LiDAR ratio [21,22], and depolarization ratio [23,24]. It was deployed in the Vipava Valley, which is not covered by any of the existing ground-based LiDAR networks, indoors, to provide as large as possible a duty time and stable operation during adverse weather conditions, including strong winds. To assure data quality and to facilitate the comparison of the retrieved optical parameters with those from other measurements, we implemented comprehensive procedures for the assessment of the uncertainties (such as polarization calibration [25,26,27], data homogenization, and error analysis [17,28]). This paper presents a detailed description of the newly-developed polarization Raman LiDAR and of the applied calibration techniques and corrections. Two observation cases are also shown to demonstrate system performance under specific selected weather conditions (during rain and in the presence of mineral dust). Observation cases demonstrating its aerosol identification capabilities were presented in [8].

## 2. System Configuration

Polarization Raman LiDAR was designed to provide simultaneously the vertical profiles of aerosol backscatter coefficients at 355 nm and 1064 nm, extinction coefficient at 355 nm, LiDAR ratio at 355 nm, depolarization ratio (volume and particle) at 355 nm, and backscatter Ångström exponent between 355 nm and 1064 nm. It was deployed in the middle of the Vipava Valley at Ajdovščina to be able to investigate directly local aerosols originating from a number of different sources. Local sources are predominantly anthropogenic, such as wood burning and traffic emissions, while remote sources are predominantly natural, as the area experiences frequent long-range transport of Saharan dust and sea-salt.

**Figure 2:**
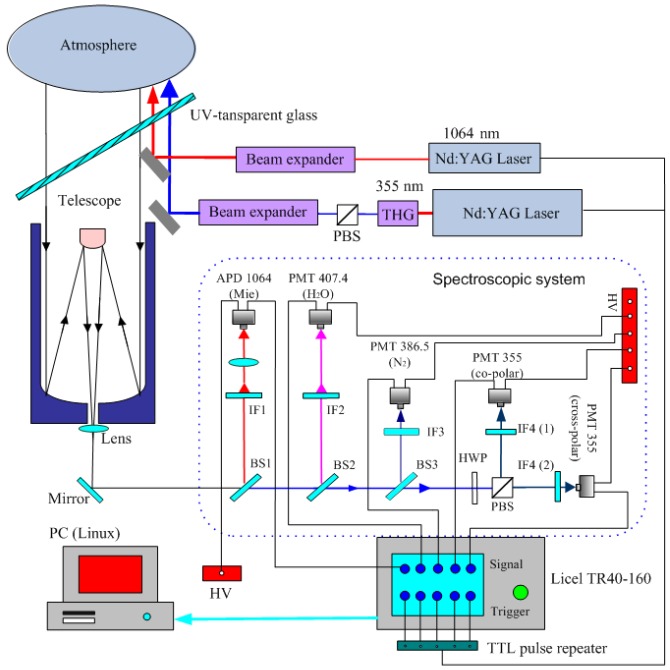
Schematic overview of the five-channel Raman polarization LiDAR system, including the transmitters, receiver, spectroscopic filter, and data acquisition chain. IF: Interference Filter, BS: Beam Splitter, PBS: Polarization Beam Splitter, PMT: Photomultiplier Tube, APD: Avalanche Photodiode, HWP: Half Wave Plate, THG: Third Harmonic Generation, HV: high voltage power supply.

The system consisted of two independent transmitters (355 nm and 1064 nm), a receiver (telescope), a spectroscopic filter with detectors for four separate channels (vibrational nitrogen Raman signal at 386.7 nm, two Mie–Rayleigh signals at 355 nm with different polarization planes, and a Mie–Rayleigh signal at 1064 nm), and the data acquisition system, including signal amplifiers and digitizers. Although the spectroscopic filter was built to provide this option, water vapor detection capabilities using the 408.4-nm Raman channel have not yet been implemented and will be a part of a future device upgrade. A schematic view of the LiDAR system is shown in Figure 2.

In order to facilitate operation in all weather conditions, including in very strong winds, the LiDAR was deployed indoors and accessed the atmosphere through a rooftop window. To achieve an adequate maximum detectable range throughout the whole troposphere of the system in the Raman channels, a large-diameter telescope (600 mm) and a high-efficiency spectroscopic system (18%–42%, depending on the channel) was used. The main system components are listed in Table 1.

**Figure 3:**
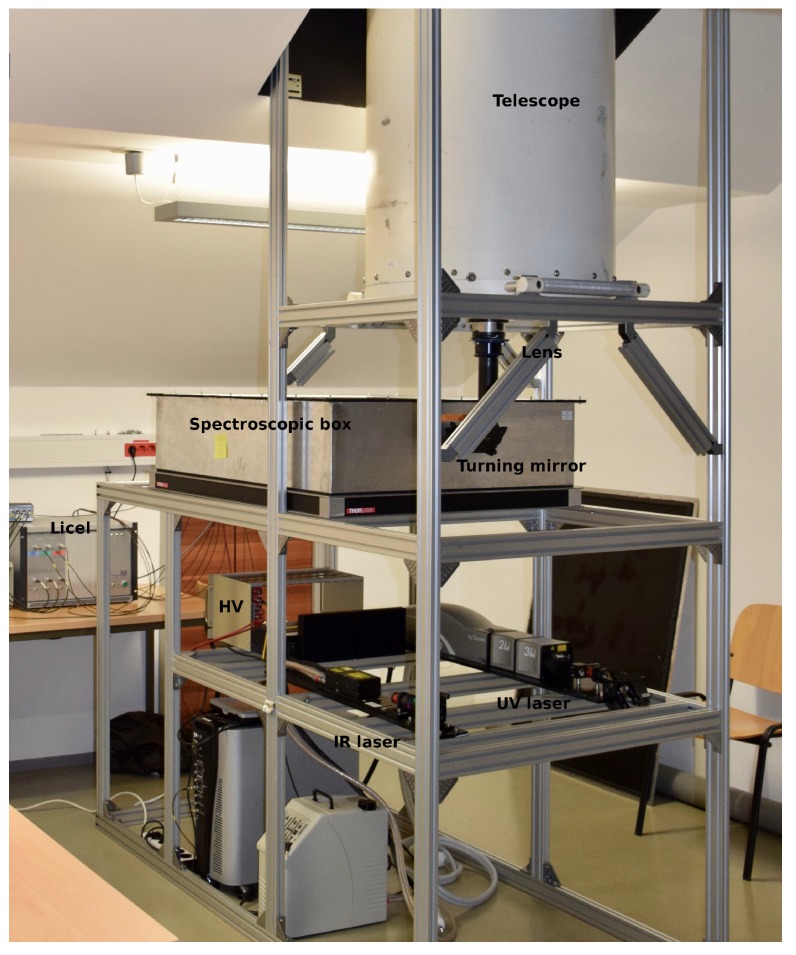
Assembled LiDAR system in its working environment at the Center for Atmospheric Research of the University of Nova Gorica in Ajdovščina. The atmosphere is accessed through a 1-m${}^{2}$ rooftop borosilicate window with >85% light transmission in UV and IR and a remotely-controlled shutter. The telescope is mounted as close to the window as possible, leaving room for the spectroscopic filter and the transmitter below.

The components were integrated by a mechanical support, which held the telescope 180 cm above the ground and very close to the window, thus suppressing the amount of stray light in the room, which could enter the telescope. This configuration also conveniently provided space for the transmitter and spectroscopic filter below the telescope. The assembled system is shown in Figure 3.

### 2.1. Transmitter

The transmitter part of the system consisted of two high-power Nd:YAG pulsed lasers (Q-smart 450 at 355 nm and Big Sky Ultra at 1064 nm), beam expanders, and turning mirrors. The Big Sky Ultra laser operating with a 20-Hz repetition rate and pulse energy of 50 mJ at 1064 nm was used to investigate Mie scattering on aerosols. The Q-smart 450 laser operating with a 10-Hz repetition rate and pulse energy of 130 mJ at 355 nm was used to investigate Raman and Mie–Rayleigh scattering. As laser pulses at 355 nm were found to be only 98.5% horizontally polarized (using a laser power-meter and a Polarization Beam Splitter (PBS) with a $\pm 1^{\circ }$ resolution mechanical rotator), a PBS with T${}_{P}$:T${}_{S}>$ 2000:1 was employed to improve the purity of the horizontally-polarized light to better than 99.99%. We also checked for possible misalignment of the horizontal polarization plane of the laser. Power loss due to the PBS was found to be minimal (1.87 mJ) when the PBS polarization plane was exactly horizontal (same as the laser), which implies that the misalignment of the horizontal polarization plane of the laser was less than $\pm 1^{\circ }$. For both lasers, the fixed full-width divergence of the beams was reduced to less than 0.2 mrad using beam expanders. A 3× beam expander was added to the 355-nm laser and a 5× beam expander to the 1064-nm laser.

### 2.2. Receiver

#### 2.2.1. Telescope

Backscattered light was gathered by an f/8 Cassegrain telescope with a 600-mm primary and 80-mm secondary aperture. A 5.08-cm diameter coupling lens, placed in front of the telescope focus, reduced the convergence of the beam from the secondary mirror. The beam converged towards the far end of the filter box, approximately 2 m from the physical end of the telescope, with a small convergence angle of only about 2.5 mrad. As a result, the beam was almost parallel throughout the spectroscopic system, providing almost the same optical conditions (same convergence angle and about a 2-cm beam size) at each detection channel. Due to the small convergence angle, the angular effects on beam splitters and filters were negligible. As the measurements were required in various weather conditions, including during strong winds, the LiDAR was installed indoors. It emitted and received signals through a rooftop window with special UV and IR-transparent borosilicate glass (Präzisions Glas & Optik, Germany) with a thickness of 5 mm and dimensions of 850 mm × 980 mm. The transmission of the window was measured to be as high as 92% for the UV at 355 nm and 95% for the IR at 1064 nm at a right angle incidence on the window pane. Due to the inclination of the roof, the incidence angle in the setup was $30^{\circ }$ with respect to the normal; thus, the actual transmissions were 85% (355 nm) and 90% (1064 nm), respectively.

#### 2.2.2. Spectroscopic Filter

**Figure 4:**
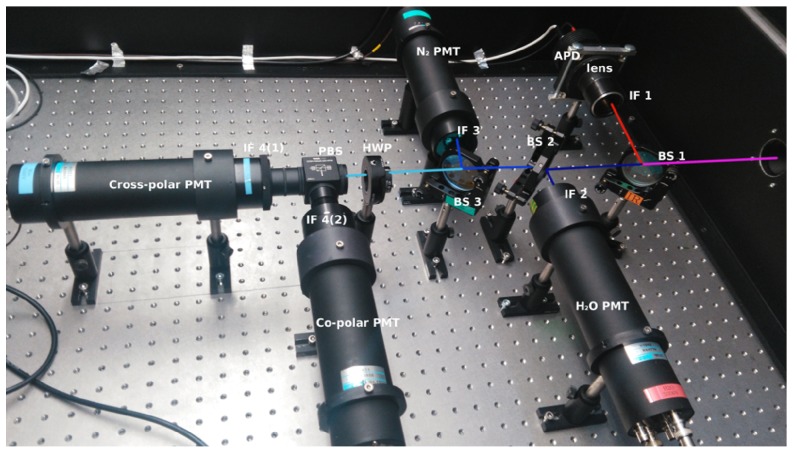
Layout of the optical components inside the spectroscopic filter with the indicated light path. The Beam Splitter (BS1), Interference Filter (IF1), aspherical lens, and Avalanche Photodiode (APD) were used for the infra-red Mie channel. BS2, IF2, and a Photomultiplier Tube (PMT) were used for the H${}_{2}$O channel. BS3, IF3, and a PMT were used for the N${}_{2}$ channel. The Half Wave Plate (HWP), Polarization Beam Splitter (PBS), two interference filters (IF4), and two PMTs were used for S and P polarization UV Mie–Rayleigh channels.

The total LiDAR return signal was separated into five channels with specific wavelengths and polarizations: the Mie–Rayleigh channel at 1064 nm, the vibrational N${}_{2}$ Raman signal at 386.7 nm, the vibrational H${}_{2}$O Raman signal at 408.4 nm, and two Mie–Rayleigh signals at 355 nm with different polarization planes. All of the spectroscopic optical components were mounted in a light-tight box (Figure 4). Dichroic beam splitters and interference filters were order made by Barr Associates https://barr-associates.com, according to the wavelength range requirements, providing high transmission or reflection efficiency. The 1064-nm Mie signal was extracted first because the components used in this channel had the lowest optical efficiency (Table 2). A Beam Splitter (BS1) reflected the infrared light to an Interference Filter, IF1, and a 25 mm-diameter, 25-mm focal length lens, which focused it onto a 1.5-mm Avalanche Photodiode (APD) detector. At the next beam splitter (BS2), the H${}_{2}$O signal was extracted using an interference filter, IF2. Beam-splitter BS3 was used to separate the N${}_{2}$ signal, which was further filtered by IF3. The light after BS3 was used for two polarized Mie channels, where the P and S polarizations were separated by a Polarization Beam Splitter (PBS) (CCM1-PBS25-355-HP, Thorlabs, www.thorlabs.com) and filtered by two interference filters of the same type (IF4). The specifications of all optical components used in the system are summarized in Table 2. The total optical efficiency for individual channels, including both the receiver and the spectroscopic filter, was estimated to be 18.3% for the infrared Mie channel, 42.9% for the H${}_{2}$O channel, 41.7% for the N${}_{2}$ channel, 33.5% and 28.7% for the P and S polarization Mie–Rayleigh channels. To verify that the 2.5-mrad full convergence angle of the received light did not hinder the performance of individual channels, we estimated the shift of the central wavelength of the interference filters [29]. The shift was found to be less than 0.002 nm for the UV and less than 0.006 nm for the IR signals, which had a negligible effect on their extraction.

### 2.3. Detectors and DAQ System

Back-scattered light was converted into electrical signals by an Avalanche Photodiode (APD) (EG&G C30954/5E, URS Corporation, San Francisco, CA, USA) in the IR range and by Photomultiplier Tubes (PMT) in the UV range. Two H1949-50 (Hamamatsu, Japan) PMTs operating at 2500 V were used in the Raman channels, and two H2341-50 (Hamamatsu, Japan), operating at 3000 V were used in Mie–Rayleigh channels. The outputs of the PMTs and the APD were amplified and digitized by transient recorders (Licel TR40-160, Licel GmbH, Germany)), which provided both analog and photon counting detection chains. For the analog detection chain, the signal was amplified according to the selected input range and digitized by a 12-bit A/D converter at 40 MHz. A hardware adder was used to write the summed signal into a 24 bit-wide RAM. Simultaneously, the signal was amplified in the Photon Counting (PC) detection chain, as well. Every photon event above the selected threshold voltage was detected by a 250-MHz discriminator, which had 64 different discriminator levels and two different settings of the preamplifier. Then, the PC signal was written to a 16 bit-wide summation RAM, which allowed averaging of up to 4094 acquisition cycles. Both detection chains provided a range resolution of 3.75 m and a maximum detection range of 61.4 km. Transient recorders were connected to a Linux-based Data acquisition (DAQ) computer via an Ethernet link. The system used our own DAQ software with the graphical interface for the detectors, digitizer, and laser control, based on C++ code and the Linux ROOT (https://root.cern.ch/) package, which allowed for fully-automated and remote operation. It was able to store both the system parameters and the digitized LiDAR return trace into tree-structured ROOT native binary files. The interface of the DAQ software also provided real-time LiDAR return information to monitor data quality [25].

## 3. Receiver Optimization and Corrections

To provide the optimal operation of the device, such as accurate determination of the backscatter return signal and in particular of the depolarization ratio, calibration of the polarization channels, overlap correction, trigger delay determination, and Rayleigh fit were performed.

### 3.1. Polarization Correction

To be able to use the depolarization information for aerosol type identification, the depolarization ratio must be corrected for different gains of the detectors in the two polarization channels (P${}^{\|}$ and P${}^{⊥}$), as well as for any geometrical misalignment between the polarization plane of the polarized transmitter and polarization planes in the spectroscopic filter with respect to the incident plane of the PBS. Both gain ratio and misalignment angle corrections were made using a Half Wave Plate (HWP) installed in front of the PBS for the polarization channels [26,30,31,32]. Polarization-relevant systematic parameters of misalignment angles, retardances, and diattenuations in the emitter and receiver systems (Table 2) were further considered to reduce the systematic uncertainty of the depolarization ratio. The alignments between the polarization plane of the transmitter and polarization planes of the $P^{\|}$ and $P^{⊥}$ channels in the receiver were accomplished by measuring their LiDAR returns. Backscattered light from the telescope was fed into the Polarization Beam Splitter (PBS) through a HWP at a number of set angles φ, which distributed known amounts of cross-polarized $P_{S}$ and co-polarized $P_{P}$ light into the corresponding channels. The PBS was oriented so that the reflection and transmission were $R_{P}=99.5$% and $T_{P}=0.5$% for horizontal polarization and $R_{S}=5$% and $T_{S}=95$% for vertical polarization. The signals observed in the two detection channels after the HWP and the PBS can be described as [26]:

(1) $$\begin{array}{c} P^{⊥}\left(\varphi \right)=P_{P}\sin^{2}\left(\varphi \right)+P_{S}\cos^{2}\left(\varphi \right),\\ P^{\|}\left(\varphi \right)=P_{S}\sin^{2}\left(\varphi \right)+P_{P}\cos^{2}\left(\varphi \right). \end{array}$$

**Figure 5:**
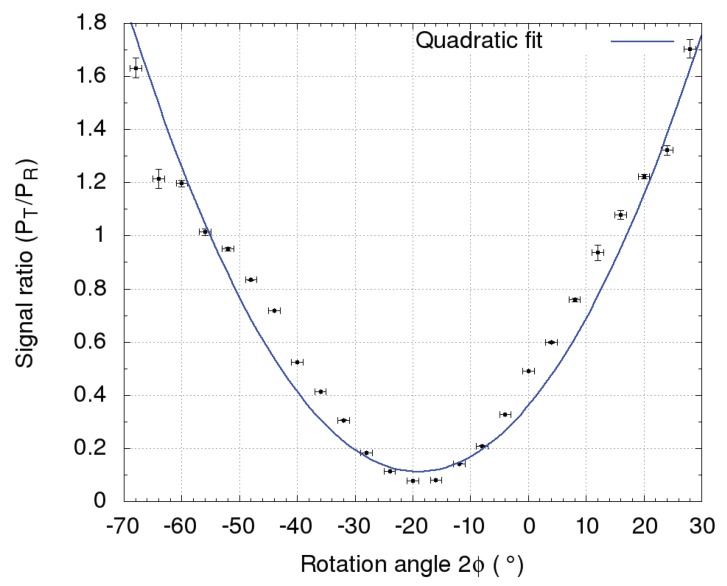
The signal ratio between $P^{\|}$ and $P^{⊥}$ with the corresponding rotation angle of the HWP. The minimum of the quadratic fit to the data points was used to determine the offset angle of the polarization plane to be $-18\pm 1^{\circ }$, where the HWP was rotated for $\phi =-9\pm 1^{\circ }$ with respect to the horizontal polarization plane of the PBS.

In calibration measurements of the LiDAR return, the HWP was rotated manually for an angle ϕ with respect to the horizontal polarization plane of the PBS using a mechanical rotator with $1^{\circ }$ angular resolution. Each of the 25 measurements, performed in consecutive 2-min intervals with a step of $2^{\circ }$ in the range of $\left[-26^{\circ }\leq \phi \leq 26^{\circ }\right]$, caused a different rotation of the LiDAR return polarization plane of 2ϕ (Figure 5).

The uncertainty of the signal ratio was estimated by the variance of the sampled return signal between 3 and 3.5 km. The angle of $\varphi_{0}=-18\pm 1^{\circ }$, yielding a minimal signal ratio ($P^{⊥}/P^{\|}$), was selected as the offset [24,26]. The relative amplification ratio *K* between two polarization channels,

(2) $$K=\frac{T_{p}+T_{s}}{R_{p}+R_{s}}\sqrt{\delta^{K}\left(-22.5^{\circ }+\varphi_{0}\right)\;\delta^{K}\left(+22.5^{\circ }+\varphi_{0}\right)},$$

was determined from measured signal fractions in the two channels, $\delta^{K}=P^{\|}/P^{⊥}$. During these measurements, the HWP was rotated $+22.5^{\circ }$ or $-22.5^{\circ }$ with respect to the offset angle $\varphi_{0}$, which corresponds to $+45^{\circ }$ or $-45^{\circ }$ polarization plane rotation of the incident light [31]. Both angles were used in the calibration to minimize the uncertainty on *K*. After calibration, HWP was set back to the offset angle $\varphi_{0}$ for regular operation, where the volume depolarization ratio $\delta_{V}$ was was retrieved [33] as:

(3) $$\delta_{V}\left(z\right)=\frac{K\left(G_{R}+H_{R}\right)-\delta^{K}\left(G_{T}+H_{T}\right)}{\delta^{K}\left(G_{T}-H_{T}\right)-K\left(G_{R}-H_{R}\right)}.$$

The depolarization parameters $G_{R}$, $G_{T}$, $H_{R}$, and $H_{T}$ were calculated by the method described in [27,33], which minimizes systematic uncertainties. The values and uncertainties of polarization-affected LiDAR parameters used in this calculation are listed in Table 3. Misalignment angles and the diattenuation of the borosilicate window were estimated by dedicated measurements, while other parameters were estimated based on the data provided by the manufacturers of the corresponding components. The window glass created a less than 4% diattenuation caused by the inclination angle and no retardance. Although an additional polarizing effect of the borosilicate window, caused by mechanical and thermal stress birefringence, could not be directly estimated, it could be the cause for the rotation of the polarization plane between the transmitter and the receiver. The remaining systematic uncertainty after polarization correction was found to be between 10% and 25%.

### 3.2. Overlap Correction

**Figure 6:**
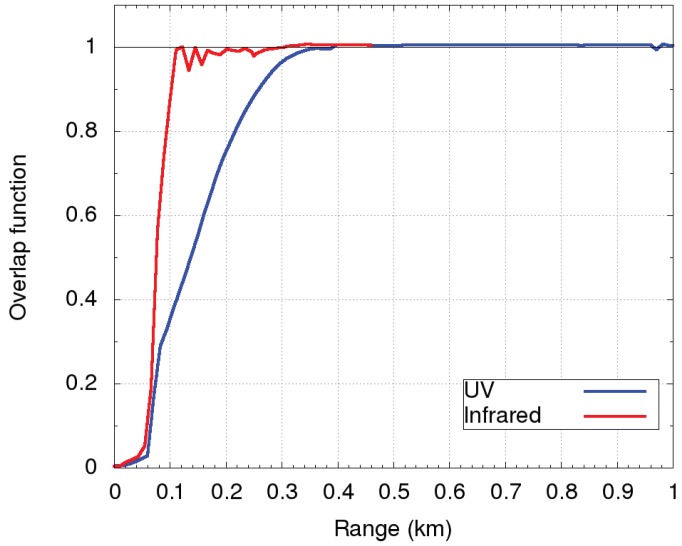
Overlap functions of the receiver and the transmitters at 355 nm (blue) and 1064 nm (red). The blue curve is based on the average of 13 nighttime measurements up to 1 km above the ground. The red curve is based on daytime measurements and obtained under the assumption of the vertical homogeneity of the aerosol concentration below 0.5 km above the ground. Both were smoothed using a running average over five consecutive range bins.

LiDAR measurements in the near range were influenced by the incomplete overlap of the field of view of the telescope and the divergence of the laser beams, emitted parallel to the optical axis of the telescope. The overlap function O(z), needed to retrieve LiDAR data at closer ranges than the complete overlap, was experimentally obtained. For the UV laser, we used the Raman method [28], which uses both Raman and Mie–Rayleigh scattering signals to retrieve backscatter and extinction coefficients. The UV channel overlap correction function was based on 13 nighttime measurements performed between 00:00 CET and 01:00 CET on days with very clear and stable weather conditions with no wind, to assure as constant a LiDAR ratio as possible. Complete overlap was achieved at the range of about 400 m. In the calculation, the LiDAR ratio was assumed to be constant within the incomplete overlap range, which was the main cause for the variation of the overlap function between different measurements. The mean overlap function with the obtained 10% variance is shown in Figure 6 (blue).

For the IR laser, the overlap function was retrieved under the assumptions of homogeneous vertical mixing and a constant LiDAR ratio within the Planetary Boundary Layer (PBL). As a result, backscatter and extinction coefficients from near the ground to the complete overlap range were taken to be constant and equal to their values just above the complete overlap. In order for the uncertainties introduced by the above assumptions to be as low as possible, LiDAR measurements were performed at times with vertically well-mixed PBL and the absence of prominent aerosol sources. In this study, daytime data (12:00 CET–12:30 CET) taken on 6 September 2017 were used, and aerosol homogeneity in the PBL above the incomplete overlap was observed (aerosol loading between 400 m and 500 m was almost constant). The main source of the uncertainty of the overlap function for IR was therefore expected to be the poorly-known LiDAR ratio (on the order of 20%), which was determined from Raman channels during the nighttime on the same day. In the IR channel, complete overlap was achieved at the range of about 200 m. The obtained overlap function is shown in Figure 6 (red).

### 3.3. Data Homogenization and Error Estimation

**Figure 7:**
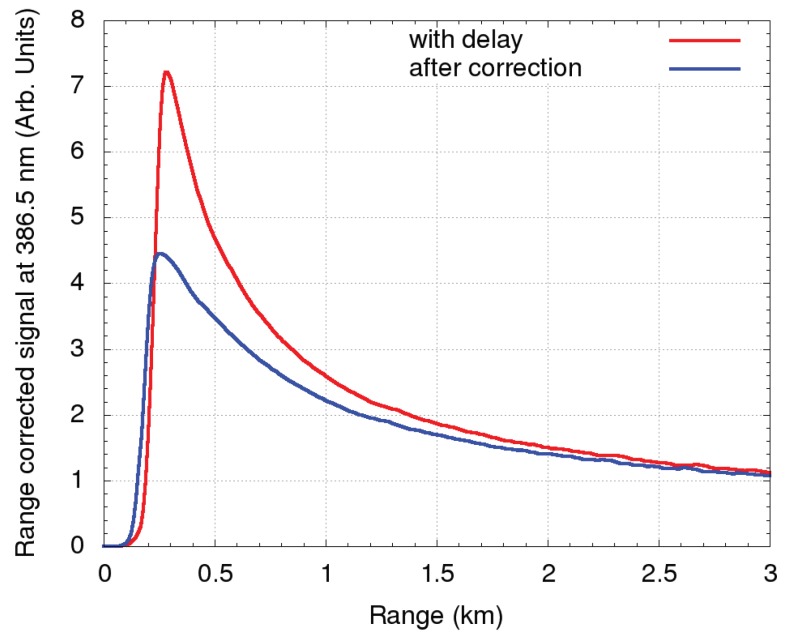
Range square corrected signal from the analog detection chain of the N${}_{2}$ channel. The red curve represents the signal with the trigger delay. The blue curve represents the signal after trigger delay correction for 52.5 m.

Instrumentation-related systematic uncertainties, originating from the laser tilt, telescope misalignment, angular dependence of the transmission of interference filters, spatial inhomogeneity of the detector sensitivity, as well as uncertainty of trigger due to the delays of individual transient recorders and dead time of the PMTs [17], can induce signal distortions, which are independent of the state of atmosphere. The near range signal up to 2 km can be significantly distorted by trigger delay (as an example, the distortion for the N${}_{2}$ signal is shown in Figure 7).

Trigger delays were measured and corrected by subtracting the obtained offset in each individual channel. Based on the system layout (Figure 3), all reflection sources in the system except the borosilicate rooftop window were eliminated, so the first data bin in the detection chain corresponded to laser pulses reflected from the window (at a known distance of about 2 m from the PMTs and APD in the receiver). The distances from the detector to the borosilicate window, obtained from both photon counting and analog detection chains in all channels, are listed in Table 4 and represent trigger delays.

Systematic uncertainties for the UV channels were estimated by fitting range-corrected backscatter signals in the aerosol-free range to attenuated molecular backscatter coefficients [17] in cloudless nighttime conditions. These were based on the molecular number density profile measured by a radiosonde at Udine, 67 km away. Range square-corrected backscatter signals were normalized to the attenuated molecular backscatter coefficient,

(4) $$\beta_{attn}\left(\lambda_{r},z\right)=\beta_{m}\left(\lambda_{r},z\right)\exp\left[-\int_{0}^{z}\left\{\alpha_{m}\left(\lambda_{0},z^{\prime }\right)+\alpha_{m}\left(\lambda_{r},z^{\prime }\right)\right\}dz^{\prime }\right],$$

using a Rayleigh fit in an aerosol-free range with a good LiDAR signal-to-noise ratio. Both $\beta_{m}\left(\lambda_{r},z\right)$, which represent molecular backscatter coefficients at range *z* and the wavelength of a selected channel, and the molecular extinction coefficient $\alpha_{m}$ were obtained using molecular number density [34]. The normalized range-corrected signal was obtained by fitting with $P_{Mie}\left(\lambda_{0},z\right)$ at the reference range $z_{min}$–$z_{max}$ as:

(5) $$z^{2}P_{norm}\left(\lambda_{r},z\right)=z^{2}P\left(z\right)\frac{\sum_{z_{min}}^{z_{max}}\beta_{attn}\left(\lambda_{r},z\right)}{\sum_{z_{min}}^{z_{max}}z^{2}P\left(\lambda_{r},z\right)}.$$

**Figure 8:**
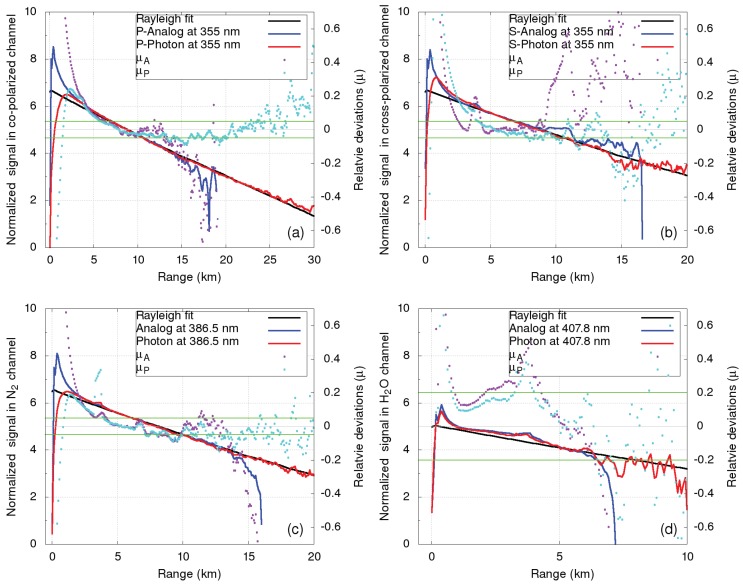
Rayleigh fit (black) to the normalized range square-corrected signals from the analog (blue) and photon counting (red) detection chains for the UV signals at 24:00 CET on 14 January 2019. Pink and light blue dots indicate deviations from the attenuated molecular backscatter coefficients calculated from the radiosonde data at Udine. Horizontal green lines indicate the individual deviations. (**a**) LiDAR signal in the $P^{\|}$ channel at 355 nm, (**b**) LiDAR signal in the $P^{⊥}$ channel at 355 nm, (**c**) LiDAR signal in the N${}_{2}$ channel at 387.5 nm, (**d**) LiDAR signal in the H${}_{2}$O channel at 407.8 nm.

After dead time correction of the PMTs in the photon counting detection chain, both analog and photon counting signals were normalized and merged in order to extend the detectable range. In the aerosol-free range, good agreement was found between normalized LiDAR signals in all UV channels and the attenuated molecular backscatter coefficients based on radiosonde data (Figure 8). In commissioning, systematic uncertainties of the backscatter signals in the UV Mie–Rayleigh and Raman N${}_{2}$ channels were found to be less than 5% for the range below 13 km and about 20% for the range below 30 km (Figure 8). The uncertainty in the H${}_{2}$O channel was found to be larger than 20%, while the uncertainty in the IR channel could not be evaluated using the Rayleigh fit due to the very low signal-to-noise ratio in the aerosol-free range.

As the typical uncertainty for the retrieval of backscatter and extinction coefficients at 355 nm in nighttime measurements using the Raman method is about 5% due to the uncertainties of the applied temperature and pressure profiles [21], the total systematic uncertainty of the retrieved backscatter coefficients at 355 nm was about 10%. The uncertainty of the LiDAR ratio, combining the uncertainties of both the backscatter and the extinction coefficients, was about 15%. Backscatter coefficients at both 355 nm in the daytime measurements and 1064 nm in all measurements can be obtained using a single elastic channel [35], providing the signal-to-noise ratio was more than one at the reference range (usually above 5–6 km). Using this method, the uncertainties of backscatter coefficients can be as low as 10% [36,37] in the case of a known predominant aerosol type; however, it is generally larger due to large uncertainty of the assumed LiDAR ratio in a more complex case of inhomogeneous vertical aerosol loading.

The Volume Depolarization Ratio (VDR) includes both molecular and aerosol contributions [27] and depends on the aerosol concentration, aerosol type, and wavelength used. The value of the molecular depolarization ratio generally ranges from about 0.004–0.04 at 355 nm, depending on atmospheric temperature, as well as on the receiver parameters. The final choice for the atmospheric molecular depolarization ratio range is from 0.008–0.01 (273 K) according to [38]. The method adopted for the VDR uncertainty analysis [27,33] corrects for the polarization-related systematic uncertainties (Equation (3)). The uncertainty of the VDR, which includes the assumed systematic uncertainties of both the $P_{\|}$ and $P^{⊥}$ channels [8], was found to vary between 10–15% and 20–25%. The uncertainty of the Particle Depolarization Ratio (PDR) depends on the uncertainties of the VDR and the uncertainties of the molecular depolarization ratio, the molecular backscatter coefficient, and the particle backscatter coefficient, which need to be estimated separately [31]. Combined, they add up to a total uncertainty of the PDR being about 10% higher than that of the VDR.

## 4. Performance of the LiDAR System

Commissioning of the new LiDAR system started in August 2017. To demonstrate its observational capabilities, we focused both on the detection of aerosol structures and aerosol properties, as well as on the characterization of aerosol types using aerosol type-dependent LiDAR retrievables (LiDAR ratio and particle depolarization ratio) under various weather conditions. In all measurements, LiDAR data were sampled with a time resolution of 1 s and range resolution of 3.75 m. Two commissioning cases focusing on aerosol properties are presented in order to demonstrate system performance. Additional detailed aerosol studies focusing on aerosol identification were given in [8].

### 4.1. Cloud Properties

**Figure 9:**
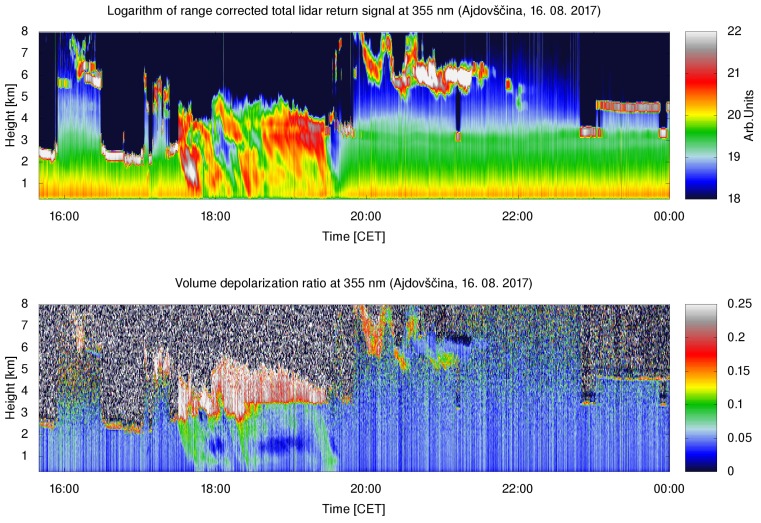
Temporal variation of the aerosol distribution over Vipava Valley on 16 August 2017 in terms of the 355-nm range-corrected LiDAR signal (top) and the volume depolarization ratio between the two polarized Mie–Rayleigh return channels (bottom). The data were resampled to a range resolution of 11.25 m. The complete overlap range was above 300 m.

**Figure 10:**
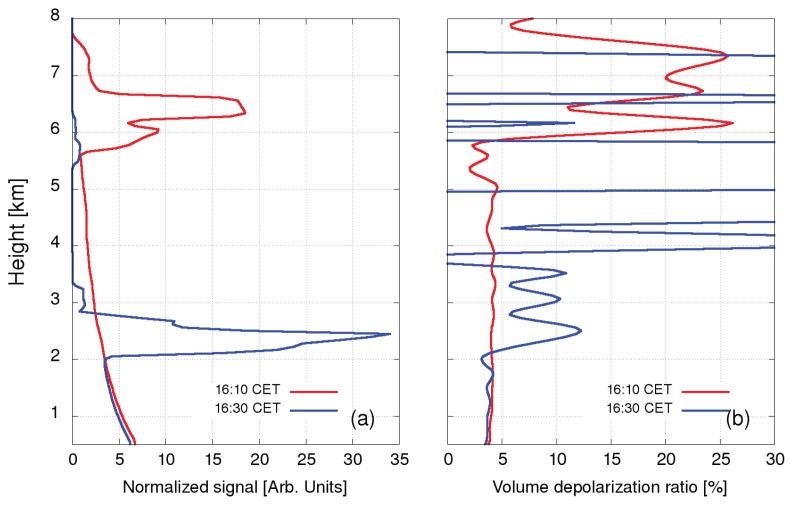
Normalized aerosol attenuated backscattering coefficients (**a**) and Volume Depolarization Ratio (VDR) (**b**) on 16 August 2017, at 16:10 CET and 16:30 CET. Peaks in the normalized attenuated backscattering coefficients determine the height of the cloud layers (6.5 km and 2.5 km). The corresponding VDR values are 0.25 and 0.15.

The first test case was aimed at verifying the system capability of the full-weather operation. The results were interpreted using a previous study of depolarization LiDAR returns in Mie–Rayleigh channels [39]. LiDAR data taken on 16 August 2017, before 15:30–17:30 CET, during 17:30–20:00 CET, and after the rain 20:00–24:00 CET are shown as a representative example (Figure 9). In the first period, the clouds were present in two separate layers, at 2.5 km and at 7 km. The VDR value of about 0.15 for the clouds in the lower layer, which is a bit higher than for a typical water cloud (0.1), but much lower than for a cloud composed of ice crystals (0.4), may be high due to multiple scattering. The VDR of the clouds in the upper layer was found to be above 0.25, which is a typical value suggested for a mixture of ice crystals and water droplets (the value between 0.25 and 0.4 indicates a mixture of water and ice crystals [39]). The depolarization ratio for aerosols below 2 km was found to be considerably lower than VDR within the clouds, where due to extremely high aerosol loading, it depends almost exclusively on aerosol type. The profiles of normalized attenuated backscattering coefficients at 355 nm and VDR profiles for both the presence of high (16:10 CET) and low (16:30 CET) clouds are shown in Figure 10. In the second period, volume depolarization ratios in both clouds increased to about 0.25, which may be the result of multiple scattering as well. Below the lower clouds, regions with raindrops with a volume depolarization ratio of about 0.1 can be identified. In the third period, a layer of scattered clouds was observed at heights between 5 and 7 km. The depolarization ratio below 0.1 indicated the presence of supercooled water, which may have been initially mixed with ice crystals [39]. The volume depolarization ratio of aerosols below 2 km decreased to about 0.04 as the rain washed out larger aerosols.

### 4.2. Aerosol Properties

**Figure 11:**
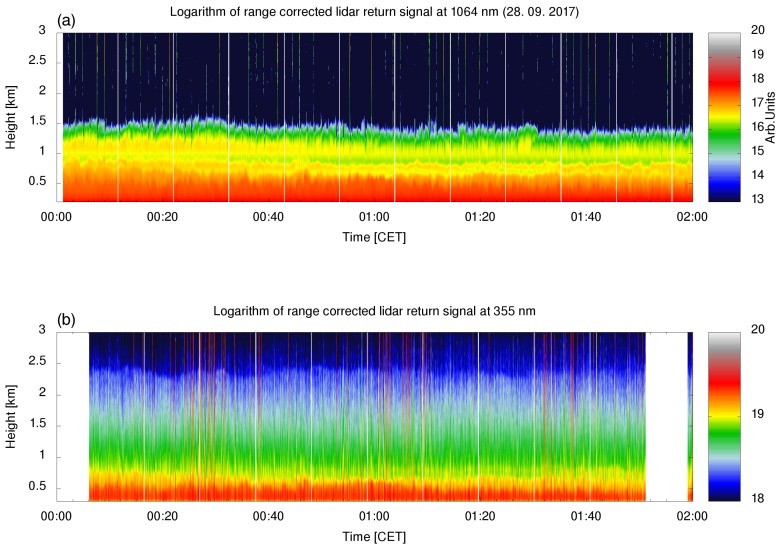
Temporal variation of the IR LiDAR return (**a**) and UV LiDAR (**b**) returns on 28 September 2017 between 00:00 and 02:00 CET shows a stable PBL with a height of about 800 m, and a residual layer with a high fraction of coarse particles up to about 1.5 km and with a low fraction of coarse particles up to about 2.5 km. The data were resampled to a 18.75-m range resolution and plotted within the complete overlap range, starting above 300 m. The heights are relative to Ajdovščina.

**Figure 12:**
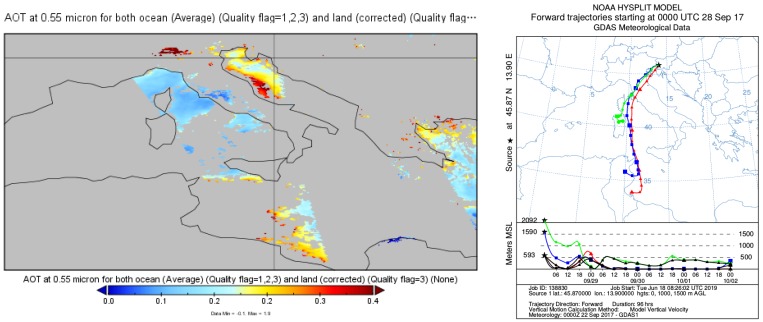
Land and ocean AOTproduct from the MODIS image of Southern Europe (including Vipava Valley) at 11:30 UTC on 28 September 2017 (left) and HYSPLIT modeled 96 h backward trajectories arriving over Vipava Valley at 00:00 CET (UTC+1) on 8 September 2017 (right). Both support the presence of aerosols from North Africa in the lower troposphere above the Vipava Valley.

**Figure 13:**
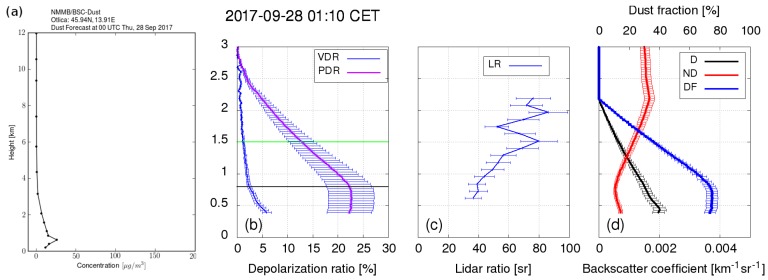
(**a**) The NMMB-BSC-DUSTmodel forecast of mineral dust concentration and its vertical distribution over the Vipava valley at 01:00 CET on 28 September 2017 (https://ess.bsc.es/bsc-dust-daily-forecast), (**b**) VDR and PDR, (**c**) LRat 355 nm, and (**d**) Dust Fraction (DF) and backscatter coefficients for Dust (D), Non-dust (ND) Aerosols. (b–d) were obtained from LiDAR measurements on 28 September 2017, 01:10–01:40 CET. Error bars stand for the estimated uncertainties. The two horizontal lines (black and green) represent the top of the PBL and the height of the elevated aerosol layer.

The second test case was aimed at confirming the system’s capability to detect relevant aerosol properties that would allow aerosol type identification. Weather conditions providing long-range transport of mineral dust were chosen for this test as they provided predominant aerosols of a known type and with known properties, which then were used to assess the LiDAR aerosol identification capability. LiDAR data were taken during an almost cloudless night from 00:00 CET–02:00 CET on 28 September 2017 (Figure 11), which followed a week of very low average daily PM${}_{10}$ concentrations in the Vipava Valley (Slovenian Environmental Agency, https://www.prowork-bb.si) ranging from 4.6 μg/m${}^{-3}$–22.2 μg/m${}^{-3}$. The daily PM${}_{10}$ average value of 28.7 μg/m${}^{-3}$ and hourly average (at 01:00 CET) of 30.6 μg/m${}^{-3}$ on 28 September 2017 therefore indicated an aerosol source that was not present in the previous days. The DREAMmodel [40], the MODIS (NASA MODIS land and ocean products, http://terra.nasa.gov/data/modis-data) satellite image, and the HYSPLIT model [41] all suggested that this additional source was long-range transport of mineral dust from North Africa (Figure 12 and Figure 13a). As the mineral dust concentration on 28 September 2017 at 01:00 CET predicted by DREAM was about 25 μg/m${}^{-3}$, we expected to observe a mixture of aged mineral dust and carbonaceous aerosols from local combustion sources within the PBL.

Temporal evolution of the LiDAR signals showed stable stratified atmospheric conditions with a stable PBL extending to about 800 m, a residual layer with a high fraction of coarse particles up to about 1.5 km, and a residual layer with a low fraction of coarse particles (combustion aerosols) up to about 2.5 km (Figure 11). The optical properties of the aerosols with their vertical distributions were investigated between 00:10 and 01:40 CET. The VDR gradually decreased with height from its maximum value within the PBL to a value close to the molecular depolarization ratio above 2.5 km, where the aerosol loading was very low. LRand PDR were almost constant at heights between the beginning of the full LiDAR overlap and the top of the PBL with values of 40 sr and 22%, which indicated the presence a large fraction of mineral dust [23,31]. Above the PBL, LR was increasing and the PDR decreasing with height, which implied a higher fraction of combustion aerosols in the residual layer with a larger absorption and smaller size [42,43]. Dust fraction and dust backscatter coefficient contributions to the total backscatter coefficient (Figure 13d) were estimated by adopting the values of PDR for pure mineral dust (30%) and the absence of mineral dust (5%) from [23]. The dust fraction decreased from 75% in the PBL down to zero throughout the residual layer. While the backscatter Ångström exponent between 355 nm and 1064 nm can be obtained using this LiDAR system [8], in this commissioning test case, the IR signal was used to identify aerosol layers only. Very low values of the IR LiDAR return and a prohibitive signal-to-noise ratio in the aerosol-free region did not allow for the retrieval of the backscatter coefficient at 1064 nm.

## 5. Conclusions

Design features of the automated polarization Raman LiDAR in Ajdovščina allowed us to use it as a stand-alone device for high duty time investigation of atmospheric properties. The performed system optimization and calibration ensured comparability of its data to measurements from large-scale LiDAR networks (i.e., EARLINET). In the commissioning, we demonstrated the functionality of the system, as well as assessed its performance. Systematic uncertainties of the retrieved LiDAR return signals were estimated to be about 5%, taking into account all uncertainty sources. The uncertainties of the measured quantities related to aerosol properties were reduced by system optimizations and by applying corrections where feasible. LiDAR performance in revealing aerosol properties and structures was investigated in two commissioning test cases. These tests demonstrated that a combination of the measured aerosol optical properties allowed for aerosol identification, which suggested that the device can be used as a stand-alone tool for aerosol typing in non-stable atmospheric conditions. The system is now entering regular operation and will provide long-term monitoring data for the investigation of aerosol sources and aerosol transport in complex terrain in a variety of atmospheric conditions, as well as for the investigation of atmospheric structures and processes in Bora wind events.

**Funding:** This research was funded by the Slovenian Research Agency (Grant Number P1-0385) and the Fulbright Scholar program.

References

## Figures and Tables

**Table 1:** Main components of the polarization Raman–Mie LiDAR. PBS: Polarization Beam Splitter, PMT: Photomultiplier Tube, APD: Avalanche Photodiode.

Transmitter		
Laser	Q-smart 450	Big Sky Ultra
Wavelength	355 nm	1064 nm
Pulse duration	5 ns	8 ns
Laser energy	130 mJ	40 mJ
Beam diameter	6.5 mm	3 mm
Repetition rate	10 Hz	20 Hz
Divergence	0.5 mrad (maximum)	0.7 mrad
Beam expander (T >90%)	3×	5×
PBS (T${}_{P}$/T${}_{S}$)	2000:1	/
steering mirror (T)	>99%	>99%
**Receiver**		
	2× Hamamatsu PMT H1949-50	
**Detectors**	2× Hamamatsu PMT H2341-50	
	1× EG&G APD C30954/5E	
**Data acquisition**		
Transient recorder	5× Licel TR40-160	
Data storage and processing	C++ code/ROOT under Linux	

**Table 2:** Specifications of the components used in the spectroscopic system. The beam splitter (BS1), interference filter (IF1), and aspherical lens were used for the infra-red Mie channel. BS2 and IF2 were used for the H${}_{2}$O channel. BS3 and IF3 were used for the N${}_{2}$ channel. HWP, PBS, and two interference filters (IF4) were used for S and P polarization UV Mie–Rayleigh channels. T${}_{P/S}$: P/S polarization transmittance, R${}_{P/S}$: P/S polarization reflectance, WL: Wavelength.

Cassegrain Telescope		Borosilicate Window	
Diameter 600 mm	f/8	Size 1 m${}^{2}$	thickness 5 mm
collimator lens	folding mirror	T >85%/90% (355/1064 nm)	inclination 30${}^{\circ }$
**Spectroscopic system**			
**BS 1**		**BS 2**	
T${}_{P}$/T${}_{S}$(< 650 nm)	>95.0%	T${}_{p}$/T${}_{s}$(345–395 nm)	>95.0%/90.0%
R${}_{P}$/R${}_{S}$(>650 nm)	>97.0%	R${}_{p}$/R${}_{s}$(400–415 nm)	>97.0%
**BS 3**		**PBS**	
T${}_{P}$/${}_{S}$(345–365 nm)	>95.0%/90.0%	T${}_{P}$/T${}_{S}$(355 nm)	95.0%/0.5%
R${}_{P}$/R${}_{S}$(385–395 nm)	>97.0%	R${}_{P}$/R${}_{S}$(355 nm)	99.5%/5.0%
**IF 1**		**IF 2**	
Central WL	1064 nm	Central WL	407.7 nm
Bandwidth	0.6 nm	Bandwidth	5.2 nm
Peak T	25.0%	Peak T	66.9%
**IF 3**		**IF 4**	
Central WL	386.5 nm	Central WL	355 nm
Bandwidth	4.8 nm	Bandwidth	1.0 nm
Peak T	65.0%	Peak T	55.0%
**Aspherical lens**		**HWP**	
Focal Length	25 mm	Central WL	355 nm
Diameter	25 mm	Rotation angle resolution	1${}^{\circ }$

**Table 3:** List of polarization-affected parameters for all main parts of the LiDAR system. Misalignment angles and the diattenuation of the borosilicate window (tilted for $30^{\circ }$ with respect to the optical axis of the telescope) were estimated by dedicated measurements. Other parameters were estimated based on the data provided by the manufacturers of the corresponding components, which are listed in Table 1 and Table 2.

**Transmitter**		
Laser	Polarization	$99.9^{\circ }$
	Misalignment angle	$0\pm 1^{\circ }$
Beam expander	Effective diattenuation	0±0.01
	Unpolarized transmission	90%
Steering mirror	Effective retardance	$0\pm 180^{\circ }$
Borosilicate window	Effective diattenuation	0.04±0.01
**Receiver**		
Borosilicate window	Effective diattenuation	0.04±0.01
folding mirror	Effective retardance	$0\pm 180^{\circ }$
Spectroscopic system	Effective diattenuation	0.08±0.01
	Misalignment angle	$18\pm 1^{\circ }$
HWP	Misalignment angle	$0\pm 1^{\circ }$
	$T_{p}$	0.5%
PBS	$T_{s}$	95%
	$R_{p}$	99.5%
	$R_{s}$	5%

**Table 4:** The distance corresponding to trigger delay for the analog and Photon Counting (PC) detection chain in each of the detection channels. The spatial resolution of the measurement was limited to 3.75 m by the range resolution of the transient recorder.

Trigger Delay	P-Mie	S-Mie	N${}_{2}$	H${}_{2}$O	IR-Mie
**Analog (m)**	45	45	52.5	41.25	41.25
**PC (m)**	3.75	3.75	7.5	3.75	-
